# Synuclein Proteins in MPTP-Induced Death of Substantia Nigra Pars Compacta Dopaminergic Neurons

**DOI:** 10.3390/biomedicines10092278

**Published:** 2022-09-14

**Authors:** Valeria V. Goloborshcheva, Valerian G. Kucheryanu, Natalia A. Voronina, Ekaterina V. Teterina, Aleksey A. Ustyugov, Sergei G. Morozov

**Affiliations:** 1 Institute of General Pathology and Pathophysiology, 125315 Moscow, Russia; 2 Institute of Physiologically Active Compounds, Russian Academy of Sciences, 142432 Chernogolovka, Russia

**Keywords:** synucleins, dopaminergic neuron, MPTP, knockout mice, Parkinson’s disease

## Abstract

Parkinson’s disease (PD) is one of the key neurodegenerative disorders caused by a dopamine deficiency in the striatum due to the death of dopaminergic (DA) neurons of the substantia nigra pars compacta. The initially discovered A53T mutation in the alpha-synuclein gene was linked to the formation of cytotoxic aggregates: Lewy bodies in the DA neurons of PD patients. Further research has contributed to the discovery of beta- and gamma-synucleins, which presumably compensate for the functional loss of either member of the synuclein family. Here, we review research from 1-methyl-4-phenyl-1,2,3,6-tetrahydropyridine (MPTP) toxicity models and various synuclein-knockout animals. We conclude that the differences in the sensitivity of the synuclein-knockout animals compared with the MPTP neurotoxin are due to the ontogenetic selection of early neurons followed by a compensatory effect of beta-synuclein, which optimizes dopamine capture in the synapses. Triple-knockout synuclein studies have confirmed the higher sensitivity of DA neurons to the toxic effects of MPTP. Nonetheless, beta-synuclein could modulate the alpha-synuclein function, preventing its aggregation and loss of function. Overall, the use of knockout animals has helped to solve the riddle of synuclein functions, and these proteins could be promising molecular targets for the development of therapies that are aimed at optimizing the synaptic function of dopaminergic neurons.

## 1. Introduction

The synuclein family consists of three highly homologous genes encoding proteins similar in structure: alpha-, beta-, and gamma-synuclein. Among the three representatives of the synuclein family, alpha-synuclein is the best-studied and the volume of scientific research devoted to its functions significantly exceeds the much-needed attention to the other two members altogether [1]. Despite extensive international studies of the synuclein family of proteins, their physiological functions as well as their pathophysiological role in synuclein-associated neurodegenerative diseases have not been fully resolved [2]. The question remains open whether the formation of Lewy bodies is the primary cause of Parkinson’s disease (PD) or whether it is a by-product of the activation of intracellular defense mechanisms against the ongoing debilitating neurodegenerative process.

In order to understand these fundamental questions, modern experimental science is developing new hybrid forms of parkinsonism in laboratory model animal systems. A toxic PD model that was initiated by single or multiple treatments of the neurotoxin 1-methyl-4-phenyl-1,2,3,6-tetrahydropyridine (MPTP) was actively used in knockout animals lacking one or more synucleins as well as in mice overexpressing a mutant form of the human protein [3,4,5,6,7]. In this review, we focus on current findings on the potential role of the synuclein family of proteins during the MPTP-induced death of substantia nigra pars compacta (SNpc) dopaminergic neurons (DA neurons) of the midbrain.

## 2. Synuclein Structure and Functions

Synucleins are a family of small soluble proteins that have at least five amino acid repeats located in the N-terminal region, resulting in an alpha-helical conformation with the C-terminal region remaining unstructured [8,9,10]. In contrast to alpha-synuclein, beta-synuclein does not contain the internal hydrophobic region corresponding with the non-beta-amyloid component (NAC) peptide, which makes alpha-synuclein capable of forming aggregates [11]. The alpha-synuclein protein was first detected in the Torpedo California electric scat in 1988 [12], but was later identified as a precursor protein in the amyloid plaques of Alzheimer’s disease patients [13]. Somewhat later, beta-synuclein was isolated from the presynaptic endings of rat and bovine brains [14,15]; gamma-synuclein was found in breast cancer metastases [16], but was further isolated from the mouse trigeminal nerve [17].

All synucleins are actively expressed in nervous system tissues. High expression levels in the neocortex, hippocampus, striatum, and cerebellum are typical for alpha- and beta-synuclein [18], but, in addition to the CNS, these proteins can also be found in blood cells, astrocytes, skeletal muscles, and the liver [11,19,20]. The first two proteins are highly represented in many structures of the brain and their levels in the spinal cord and peripheral nervous system are relatively low; the opposite is found for gamma-synuclein, with a high expression level in the motor neurons of the spinal cord and medulla oblongata, neurons of the sympathetic and parasympathetic peripheral nervous system, tumor entities, and retinal ganglion cells [9,21].

Despite independent roles in the cell, synucleins are highly homologous and have similar functions, often compensating for the dysfunction between each other. Synucleins are important for the synaptic transmission and circulation of synaptic vesicles [22,23,24,25,26,27]. Alpha-synuclein modulates the release of neurotransmitters from presynaptic terminals by binding and clustering synaptic vesicles and chaperoning the soluble N-ethylmaleimide-sensitive factor attachment protein receptor (SNARE) complex assembly by binding to the protein synaptobrevin-2 (VAMP2) [28,29] whereas beta-synuclein and gamma-synuclein modulate the synaptic vesicular binding of alpha-synuclein and thus reduce the synaptic physiological activity of alpha-synuclein [30,31] (Table 1). Moreover, in vitro and in vivo experiments have revealed that all three members of the synuclein family have chaperone activity [32,33,34].

Alpha-, beta-, and gamma-synucleins can bind to the dopamine transporter (DAT) and modulate its delivery to the synaptic membrane, thereby affecting dopamine neurotransmitter reuptake [11,31,35]. In turn, it has been shown through protein–protein interactions that alpha-synuclein can affect DAT activity and this effect is regulated by the gamma-synuclein concentration [36,37,38]. Alpha-synuclein is involved in maintaining the required level of dopamine (DA) and if its function cannot be performed due to a mutation, a vesicle degradation occurs [27]. The mechanism of this effect has multiple roots: alpha-synuclein regulates synaptic DA homeostasis [39], affects the expression of DA synthesis member genes (such as GTP-cyclohydrolase, tyrosine hydroxylase (TH), and aromatic acid decarboxylase) [40], modulates synaptic DA reuptake by binding to DAT [41], and inhibits DA release in response to repeated excitation [42,43]. Previously, there was no evidence for an interaction between beta-synuclein and TH, but it has been suggested that it may functionally overlap with alpha-synuclein [44]. Moreover, a recent study convincingly demonstrated that beta-synuclein potentiates vesicular dopamine uptake, presumably by the assembly of the TH/AADC/VMAT-2 protein complex, which is probably not functionally compensated by alpha- or gamma-synuclein [4].

Synucleins are also lipid-binding proteins capable of inducing membrane curvature and turning large vesicles into highly curved formations [11,31,45]. Moreover, gamma-synuclein regulates lipid metabolism in adipocytes and the lack of this protein has a significant impact on the energy metabolism of the whole organism [46]. In addition, alpha- and beta-synucleins prevent cell autolysis. For example, beta-synuclein possesses p53-dependent anti-apoptosis properties at low physiological concentrations, inhibiting caspase-3 activation by binding to Akt [47,48].

A number of studies have found that alpha-synuclein is able to bind to the mitochondria and even penetrate them through VDAC channels (the outer membrane metabolic channel), thus probably targeting the mitochondrial respiratory chain complexes in the inner membrane [49,50,51], but the physiological significance of this interaction remains unclear. A difference in the lipid composition of the mitochondrial membrane is a regulatory link in the affinity with the alpha-synuclein–VDAC interaction [52].

Beta-synuclein binds to metals to regulate cellular metal homeostasis, particularly chelated copper ions, which can produce free radicals and promote the formation of cytotoxic alpha-synuclein oligomers [11,53,54]. There is also a suggestion that beta-synuclein can affect the autophagic–lysosomal pathway, removing damaged or toxic protein molecules and even aggregates [55,56]. In turn, gamma-synuclein optimizes the autophagy process, which protects colon cancer cells from endoplasmic reticulum stress [57].

The ubiquitin–proteasome system that provides controlled protein degradation is extremely important for the removal of toxic oligomers and soluble protofibrillar structures formed by proteins prone to aggregation, including synucleins. All three members of the synuclein family are able to interact with proteasomes but with different efficiencies. For alpha-synuclein, the interaction depends on the degree of its aggregation [58,59]. Monomeric beta-synuclein also has a low inhibitory effect on 20S and 26S proteasome complexes, but monomeric gamma-synuclein inhibits ubiquitin-independent proteolysis much more effectively. Interestingly, beta-synuclein acts as a negative regulator of alpha-synuclein in these processes [60].

Gamma-synuclein is involved in the stabilization of the cell cytoskeleton [61]. Although alpha-synuclein is capable of interacting with a few components of the cytoskeleton—in particular, with tubulin—the putative effects of alpha-synuclein on its polymerization are not clear [62]. In the lysates of cancer cells, gamma-synuclein was found both in the cytosolic fraction and in the cytoskeleton fraction and the role of gamma-synuclein in stabilizing the neurofilament network in neurons was also revealed [63].

Interestingly, several studies have shown a modulating role of alpha-synuclein in the formation of populations of the SNpc DA neurons of the midbrain. Alpha-synuclein takes part in the maturation of SNpc DA neurons whilst the development of the adjacent similar anatomical structure, the ventral tegmental area (VTA), proceeds independently [64]. In turn, one of the possible roles of synucleins is considered to be participation in the regeneration of damaged neural tissues. It was found that the concentration of alpha- and beta-synucleins (gamma-synuclein-less) was significantly increased around damaged neural endings [65,66]. Hence, the link between synucleins and neurodegeneration can be explained not only by pathological aggregation and its induced toxicity, but also by the loss of normal function. Disruptions in the structure, intracellular localization, and compartmentalization of the synuclein family of proteins result in pathological conditions called synucleinopathies.

## 3. Parkinson’s Disease Is a Form of Synucleinopathy

Parkinsonian syndrome (or parkinsonism) is a neurological condition with a multifactorial etiology caused by a disorder in the extrapyramidal system of the brain. Parkinsonism is clinically characterized by a triad of signs (bradykinesia, rigidity, and tremor) and it has additional motor and non-motor pathological manifestations. The debut of the disease usually occurs between the ages of 65 and 70, with less than 5% of cases in patients younger than 45 [67,68].

According to worldwide statistics, the prevalence of parkinsonism in the general population ranges from 100 to 200 cases per 100,000 people, with an annual increase of 15 cases per 100,000 people [69]. In reality, these figures are underestimated due to the low detection rate at the initial stages of the disease and difficulties in the differential diagnosis of various extrapyramidal pathologies burdened with a PD-like set of symptoms.

Parkinson’s disease (PD) is the most common form of parkinsonian syndrome and it is etiopathogenetically designated as primary or idiopathic parkinsonism. However, there are other clinical forms of neurodegenerative diseases to be considered. These include progressive supranuclear palsy (Steele–Richardson–Olszewski syndrome), Huntington’s chorea, and corticobasal degeneration (CBD) as well as secondary drug-induced toxic parkinsonism and many others [70,71]. These diseases can be differentiated on the basis of key clinical features as well as a clear understanding of the pathogenetic mechanisms underlying PD, which is crucial for the diagnosis, treatment, and prognosis of the neurodegenerative process in the extrapyramidal system.

Pathophysiologically, PD is characterized by the degeneration of dopaminergic neurons in the substantia nigra of the midbrain due to the cytotoxic aggregation and formation of cytoplasmic inclusions—Lewy bodies (LBs)—resulting in a dopamine deficiency in the striatum and in other associated structures of the basal ganglia [72,73,74]. LBs contain aggregated forms of the alpha-synuclein protein, which is also present in other neurodegenerative disorders, including multiple system atrophy, dementia with Lewy bodies [75,76], Hallervorden–Spatz disease, and many others that are collectively referred to as “synucleinopathies” [77]. Although a small percentage of patients with PD have a monogenic form of the disease (LRRK2, parkin, etc.), in most cases the disorder is sporadic with an unknown etiology. Normally, alpha-synuclein is present in several states, such as monomeric, dimeric, oligomeric, and fibrillar forms. However, alpha-synuclein oligomers exert the most toxic effects on DA neurons [78]. An increased concentration of alpha-synuclein oligomers was found in the substantia nigra [79,80], cerebrospinal fluid [81], and blood [82] of PD patients. The intranasal administration of oligomeric forms of alpha-synuclein to C57BL/6J mice caused PD-like symptoms [83]. These data all suggest that the oligomeric form of alpha-synuclein has a pathogenetic significance in the development of PD. However, the exact mechanisms of the involvement of alpha-synuclein oligomers in the death of nigrostriatal dopaminergic neurons are currently unknown.

A joint injection of MPTP and alpha-synuclein oligomers into the striatum of C57BL/6J mice resulted in the activation of astrocytes and microglia in the substantia nigra and increased the loss of nigral TH+ neurons and the development of motor deficits in animals to a greater extent than MPTP-only treatments. These results indicate that alpha-synuclein oligomerization induces a neurotoxic effect on DA neurons in SN [6]. Activated microglia secrete proinflammatory cytokines IL-1β, IL-6, IL-10, interferon gamma (IFN-γ), and tumor necrosis factor-α (TNF-α). These secretions activate the nuclear transcription factor NF-kB, triggering core apoptosis and inducible NO synthase (iNOS), leading NO and other ROS and cyclooxygenase-2 (COX2) to increase the formation of prostaglandin E2. The presence of these pathogenic factors eventually causes the death of SNpc DA neurons [84]. Thus, it is crucial to use various models of parkinsonism—including laboratory animals such as transgenic mice with an overexpression of a mutant form of human alpha-synuclein (A53T; A30P), toxin-induced models (6-hydroxydopamine (6-OHDA), MPTP, and reserpine), and knockout mice lines with a depletion of Parkin/Park genes (Pink-1, DJ-1, and synuclein family proteins)—in order to fully understand the mechanisms of PD pathogenesis.

### 3.1. Toxic Animal Modeling of Parkinsonism Using MPTP

The toxic modeling of parkinsonism with MPTP was proposed at the end of the 20th century. Dr. Langston discovered clinical PD symptoms in addicts of “synthetic heroin”, which contained MPTP as one of its byproducts [85]. The discovery of MPTP-induced parkinsonian syndrome provoked a number of scientific studies worldwide that were aimed at determining the pathophysiological mechanisms underlying parkinsonism and it raised the disciplines of neurochemistry and neurobiology to a new level. Thus, MPTP was found to cause the extensive selective death of dopaminergic neurons in the substantia nigra [86]. The results of biochemical studies and an analysis of the cytoarchitectonics of SNpc revealed a decrease in the dopamine content in the striatum and a decrease in the number of nigrostriatal DA neurons in various MPTP-treated animals, including monkeys [87], dogs [88], cats [89], mice [64], and even frogs [90]. A local neurodegeneration caused by a single injection of MPTP at relatively low doses (5–10 mg/kg for dogs and cats; 30 mg/kg for mice) resulted in symptoms (hypokinesia, muscle rigidity, and tremor) that were typical for idiopathic parkinsonism. Yet, not all laboratory animals are sensitive to MPTP. For example, rats, rabbits, and guinea pigs required relatively high doses of MPTP (50–70 mg/kg) in order to manifest the neurological signs of an extrapyramidal system dysfunction, which leads to the development of parkinsonism [91].

MPTP is a lipophilic compound that freely crosses the blood–brain barrier and is metabolized by MAO-B in the glial cells to 1-methyl-4-phenylpyridine in an ionic form (MPP+), which is a highly toxic final metabolite [85,92]. DA neurons in the SNpc then selectively capture MPP+ from the intercellular space using the membrane transporter DAT due to its structural similarities to the dopamine molecule [93]. MPP+ accumulates in the mitochondria where it inhibits complex I of the electron transport chain, leading to the inhibition of cellular respiration [94,95], decreased ATP production [96,97], oxidative stress [98,99], the activation of the caspase cascade [100], and, ultimately, cell death.

The MPTP-toxic model of parkinsonism induced in C57BL/6J mice is widely accepted as the primary system to study the pathogenetic mechanisms that underlie extrapyramidal system disorders and that contribute to PD as well as to develop prospective neuroprotection strategies. Over the past decades, numerous protocols have been created to model toxic parkinsonism. These protocols are grouped based on the speed and severity of the clinical signs into three main categories: “acute administration” (several MPTP doses in one day); “subchronic administration” (usually 1–2 doses a day for a 5-day period); and “chronic” administration (multiple injections for 1 month or more) [101,102].

**Figure 1:**
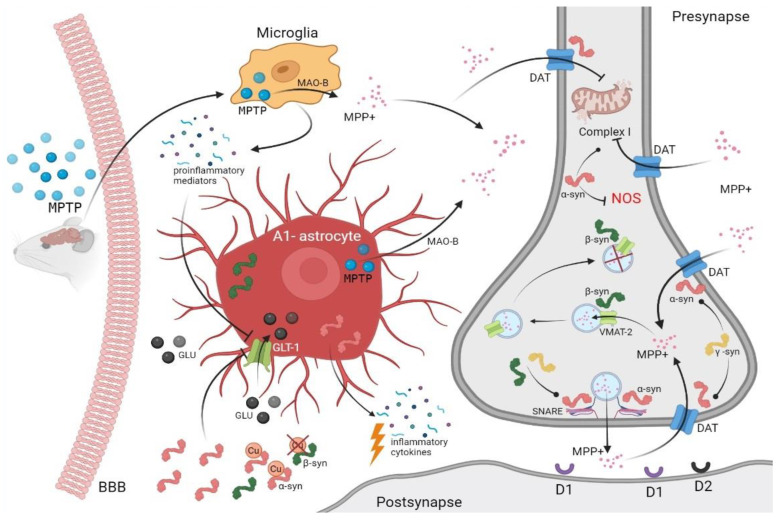
The role of synucleins in the mechanisms of SNpc DA neurons during MPTP-induced parkinsonism. Key regulatory factors include the regulatory activity of all synucleins toward the presynaptic membrane of the dopamine transporter (DAT); increased DAT/VMAT-2 ratio and SNARE assembly due to the presence of alpha-synuclein and support from other members of the synuclein family; the inability of beta-synuclein in the presence of alpha- and gamma-synucleins to potentiate VMAT-2-dependent MPP+ capture to further sequester these molecules; the involvement of alpha-synuclein in the neuroinflammatory response; and glutamate toxicity induced by glial cells. These, as well as other unexplored effects of alpha-synuclein binding and penetration into damaged mitochondria, may have a special effect on the MPTP-induced death of DA neurons. Created with BioRender.com (accessed on 2 November 2021).

As indicated earlier, synuclein family proteins are actively involved in the processes of dopamine neurotransmission in the presynaptic endings of SNpc DA neurons. The saturation of the presynaptic endings of DA neurons with the toxic end-metabolite of MPTP—1-methyl-4-phenylpyridine in an ionic form (MPP+), which has a high affinity with the plasma membrane transporter DAT—is presumably directly related to the activity of synuclein family proteins (Figure 1). Thus, the selective pathological effect of MPP+ is based on the ability of neurons to reuptake the neurotransmitter from the synaptic cleft in order to replenish the intracellular stores and form new vesicles [103] where synucleins could play a special role.

### 3.2. Synucleins and MPTP Toxicity

Dopamine is the most important signaling neurotransmitter that regulates the motor function of the entire extrapyramidal system, which is responsible for the superstructure of movements [104]. MPP+ is structurally similar to dopamine and it competes for binding sites on the presynaptic membrane of DA neurons. In toxic conditions, such as parkinsonism, DA neurons are particularly sensitive and vulnerable to the pathological effects of MPP+, which entails a series of dramatic events leading to the complete degeneration of the nigrostriatal pathway because DA neuron bodies lie in the substantia nigra of the midbrain with axons extending to the dorsal striatum. On the other hand, it is not quite clear what role synuclein family proteins play in these processes as the main representative of the family, alpha-synuclein, acts as a pathological marker of PD.

The first and subsequent studies on the effects of MPTP toxicity in alpha-synuclein-deficient animals showed surprising results: acute and chronic neurotoxin administration protocols did not have the desired effect on the death of the DA neurons of the SNpc despite lower cell counts [105,106,107,108] (Table 2). Moreover, several in vitro studies demonstrated that an overexpression of human alpha-synuclein was associated with enhanced cell death after MPP+ exposure [109,110]. MPTP administration to mice with a selective inactivation of alpha-synuclein in a few cases resulted in a dopamine deficiency and the manifestation of early clinical symptoms of a dopaminergic system dysfunction typical of the early stages of PD [7], which indirectly indicated the activation of the compensatory mechanisms of DA/MPP+ neurotransmission. It is worth emphasizing that phenotypically alpha-synuclein-knockout mice do not differ from wild-type animals [111,112]. However, decreased levels of striatal dopamine in a few lines [113,114] resulted in a reduced availability of DAT on neuronal surfaces [107] and the early debut of Parkinson-like symptoms in aging mice [114,115,116]. Although neurons manage to compensate for a lack of alpha-synuclein, this takes a toll on the restructuring of the defense systems, which, under certain conditions, can lead to the development of pathological processes, primarily in those cellular compartments where alpha-synuclein normally functions.

In turn, animals with a gamma-synuclein deficiency showed a similar response to MPTP-induced dopaminergic neurodegeneration. Here, the main feature was also the resistance of SNpc DA neurons to the toxic effect of MPTP [5,113,116]. Notably, a comparative immunoblotting analysis of the synuclein levels in the midbrain of gamma- and alpha-synuclein-knockout vs. wild-type mice showed increased levels of beta-synuclein [5,117]. This phenomenon led to a further strategy to investigate the role of synucleins in the development of MPTP resistance.

Recent studies have convincingly demonstrated that beta-synuclein is involved in optimizing the capture of dopamine and probably that of structurally similar molecules via VMAT-2 (vesicular monoamine transporter-2) [4]. Moreover, there was a loss of resistance of the DA neurons in the SNpc to MPP+, which is a toxic metabolite of MPTP, in beta-synuclein knockouts. A similar effect was observed in triple-knockout mice (triple synuclein-deficient mice), where the initial population of DA neurons in the SNpc was similar to wild-type mice [4]. In cases of alpha- and/or gamma-synuclein deficiency there was a 2.8-fold increase in the VMAT-2 density per vesicle [107], probably due to the increased presence of beta-synuclein at the presynaptic end, which was consistent with other studies [5]. However, DA neurons in the SNpc are known to be particularly susceptible to MPP+ because they have a higher DAT/VMAT-2 ratio than other brain neurons [11]. Thus, a reduced DAT transporter in the presynapse, combined with an increased VMAT-2 density in the vesicles, changed the VMAT-2/DAT ratio, leading to the utilization of toxic MPP+ molecules. Taken together, these results suggest a direct involvement of beta-synuclein in the developmental processes of the resistance of SNpc DA neurons to neurotoxins rather than the absence of alpha- or gamma-synucleins *per se*.

The potential neuroprotective properties of beta-synuclein also include the regulation of cellular apoptosis. Serine threonine kinase (Akt) is an enzyme that inhibits apoptosis by phosphorylating the Mdm2 protein that binds to p53 in the nucleus. In an experiment by Hashimoto et al., it was shown that a beta-synuclein overexpression in a rat neuroblastoma B103 cell line resulted in the resistance of these cells to the toxic action of rotenone, which, in a similar manner to MPTP, inhibits mitochondrial respiratory chain complex I. However, an Akt inhibition in this cell line resulted in the loss of neuronal resistance to neurotoxin exposure [118].

The specific damaging effect of MPTP on catecholaminergic neurons is also associated with the activation of toxic A-astrocytes, which, under the influence of proinflammatory mediators, inhibit the glutamate capture via GLT-1 and induce the production of inflammatory cytokines, leading to neuroinflammation [119]. Moreover, a disruption of the Nrf2 system in astrocytes leads to a decrease in the number of antioxidant molecules, resulting in oxidative stress. Damaged DA neurons secrete oligomeric alpha-synuclein in PD. The transfer of alpha-synuclein from neurons to astrocytes, with the subsequent accumulation and deposition in astrocytes, leads to the formation of proinflammatory cytokines and the disruption of the glutamate capture via GLT-1 [119]. Such a scenario is possible in the case of a long-term protocol of chronic MPTP administration, for which the presence of amyloid-like inclusions in both the astrocytes and DA neurons in the SNpc has been noted [120,121].

An abnormal aggregation of alpha-synuclein can increase the degree of glutamate excitotoxicity. Alpha-synuclein accumulation in astrocytes affects the glutamate transport, causing increased extracellular glutamate concentrations and excitotoxicity, further aggravating the damage to the dopaminergic neurons [122]. These data emphasize that alpha-synuclein increases the glutamate release. The concentration of alpha-synuclein itself depends on the release of activity-dependent presynaptic glutamate from the endings of the forebrain neurons [123]. In addition, the overexpression of alpha-synuclein increases the phosphorylation of N-methyl-D-aspartate (NMDA) receptors, thereby increasing the formation of NR1 and NR2B subunits and the sensitivity of NMDA receptors to developing glutamate excitotoxicity [124]. Increased levels of glutamate in the intercellular space activates glutamate NMDA receptors, leading to a calcium overload and the death of DA neurons [125,126,127]. Alpha-synuclein can also enhance glutamate excitotoxicity by accelerating α-amino-3-hydroxy-5-methyl-4-isoxazolepropionic acid (AMPA) receptor signaling [128].

The formation of reactive oxygen species (ROS) is directly involved in the pathogenesis of MPTP-induced parkinsonism [103]. It is unclear how synuclein family proteins are related to these events. It has been established that an alpha-synuclein deficiency leads to the inhibition of nitric oxide synthase (NOS), which forms another powerful oxidant, peroxynitrite (ONOO^−^), by interacting with ROS [107]. Thus, NOS activation is an important step in MPTP-induced toxicity and it can be inhibited by a targeted inactivation of alpha-synuclein. Therefore, this targeted inactivation could be a promising direction for the development of a PD therapy.

Finally, there is an assumption that alpha-synuclein specifically interacts with the mitochondria by blocking the toxic effect of neurotoxins, which have an established pathogenic action on DA neurons, leading to the development of PD [129,130]. However, this protective function of synucleins does not extend to all cells; in particular, not to differentiated DA neurons. This may imply that the cytoprotective properties of alpha-synuclein are aimed at optimizing the mitochondrial function and directly depend on the stage of cell differentiation; i.e., are linked to aging [27]. This is indirectly confirmed by studies of the role of alpha-synuclein in the maturation of SNpc DA neurons in the early postnatal developmental period [64].

## 4. Concluding Remarks and Future Directions

All proteins of the synuclein family are distributed throughout the nervous system, predominantly performing the optimization and systematization functions of various processes. Based on all the studies summarized in this review, we conclude that the differences in the sensitivity of synuclein-knockout animals compared with MPTP neurotoxin models are due to and result from the ontogenetic selection of early neurons followed by a compensatory effect of beta-synuclein, which optimizes the DA capture in the synapses. This is supported by MPTP toxicity data from synuclein-free animals with the inactivation of all three members. Compared with single alpha- or gamma-synuclein knockouts, the sensitivity of DA neurons to the toxic effects of MPTP is higher in triple-knockout animals and almost identical to the levels shown in wild-type controls, suggesting that beta-synuclein could modulate the alpha-synuclein function, preventing its aggregation and a loss of function. Thus, synucleins can be considered to be promising molecular targets for the development of therapies that are aimed at optimizing the synaptic function of dopaminergic neurons. Knockout mice lacking any of the three synuclein members could be used as a promising tool to study the mechanisms of the neurodegenerative processes of synucleinopathies such as PD.

## Figures and Tables

**Table 1:** Physiological functions of synuclein proteins.

Functions	α-syn	β-syn	γ-syn	Ref.
Neurotransmission	✔	✔	✔	[22,23,24,25,26,27]
Chaperoning	✔	✔	✔	[32,33,34]
SNARE assembly	✔	Maintenance	Maintenance	[28,29,30,31]
DAT transporter delivery to the presynapse	✔	✔	✔	[11,31,35]
Regulation of DAT transporter activity	✔	NA	Maintenance	[36,37,38]
Regulation of dopamine homeostasis	✔	**?**	NA	[27,39,40,41,42,43,44]
Potentiation of vesicular dopamine uptake	NA	✔	NA	[4]
Lipid structure or morphology changes	✔	✔	✔	[11,31,45]
Regulation of lipid metabolism	NA	NA	✔	[46]
Anti-apoptosis	✔	✔	NA	[47,48]
Mitochondrial regulation	**?**	NA	NA	[49,50,51,52]
Regulation of cellular metal homeostasis	NA	✔	NA	[11,53,54]
Regulation of the autophagic–lysosomal pathway	NA	**?**	✔	[55,56,57]
Interaction with proteasomes	✔	✔	✔	[58,59,60]
Cytoskeleton stabilization	**?**	NA	✔	[61,62,63]
Regulation of the growth of neurons in SNpc	✔	NA	NA	[64]
Regeneration of damaged neurons	**?**	**?**	**?**	[65,66]

✔: involved; NA: not available; **?**: hypothesis.

**Table 2:** Main phenotypic changes in synuclein-knockout animals before and after MPTP injections.

Effect	MPTP *	α-syn KO	β-syn KO	γ-syn KO	αβγ-syn KO
Clinical manifestation	–	**≈**	**≈**	**≈**	**≈**
	**+**	✔	NA	NA	✖
Striatal dopamine	–	**≈**	▼	**≈**	▼
	**+**	▼	▼	NA	▼
DAT expression	–	▼	NA	NA	NA
	**+**	NA	NA	NA	NA
SNpc neurons	–	▼	**≈**	▼	**≈**
	**+**	resistant	▼	resistant	▼

**≈**: similar to wild-type animals; ✔: presence; ✖: absence; ▼: decrease; NA: not available; *: subchronic MPTP administration.

## Data Availability

Not applicable.

## Abbreviations

PD	Parkinson’s disease
DA	Dopamine
NF-kB	Nuclear factor kappa-light-chain-enhancer of activated B cells
DA neurons	Dopaminergic neurons
MPTP	1-methyl-4-phenyl-1,2,3,6-tetrahydropyridine
MPP+	1-methyl-4-phenylpyridine in ionic form
SNpc	Substantia nigra pars compacta
NAC	Non-beta-amyloid component
CNS	Central nervous system
SNARE	Soluble N-ethylmaleimide-sensitive factor attachment protein receptor
VAMP-2	Protein synaptobrevin-2
VMAT-2	Vesicular monoamine transporter-2
DAT	Dopamine transporter
TH	Tyrosine hydroxylase
VDAC	Voltage-dependent anion channels
VTA	Ventral tegmental area
CBD	Corticobasal degeneration
IL-1β	Interleukin-1β
IL-6	Interleukin-6
IL-10	Interleukin-10
IFN-γ	Interferon gamma
TNF-α	Tumor necrosis factor-α
MAO-B	Monoamine oxidase B
iNOS	Inducible NO synthase
NO	Nitric oxide
ONOO	Peroxynitrite
COX2	Cyclooxygenase-2 enzyme
NMDA	N-methyl-D-aspartate
AMPA	α-Amino-3-hydroxy-5-methyl-4-isoxazolepropionic acid
ROS	Reactive oxygen species
